# Comparison of standard and over-the-head method of chest compressions during cardiopulmonary resuscitation - a simulation study

**DOI:** 10.1186/s12873-019-0292-8

**Published:** 2019-11-26

**Authors:** Michał Ćwiertnia, Marek Kawecki, Tomasz Ilczak, Monika Mikulska, Mieczysław Dutka, Rafał Bobiński

**Affiliations:** 1 Department of Emergency Medicine, Faculty of Health Sciences, University of Bielsko-Biala, Willowa 2, 43-309 Bielsko-Biala, Poland; 2 Department of Biochemistry and Molecular Biology, Faculty of Health Sciences, University of Bielsko-Biala, Willowa 2, 43-309 Bielsko-Biala, Poland

**Keywords:** Emergency medicine, Sudden cardiac arrest, Cardiopulmonary resuscitation, Advanced life support, Heart massage

## Abstract

**Background:**

Maintaining highly effective cardiopulmonary resuscitation (CPR) can be particularly difficult when artificial ventilation using a bag-valve-mask device, combined with chest compression have to be carried out by one person. The aim of the study is to compare the quality of CPR conducted by one paramedic using chest compression from the patient’s side with compression conducted from the ‘over-the-head’ position.

**Methods:**

The subject of the study were two methods of CPR – ‘standard’ (STD) and ‘over-the-head’ (OTH). The STD method consisted of cycles of 30 chest compressions from the patient’s side, and two attempts at artificial ventilation after moving round to behind the patient’s head. In the OTH method, both compressions and ventilations were conducted from behind the patient’s head.

**Results:**

Both CPR methods were conducted by 38 paramedics working in medical response teams. Statistical analysis was conducted on the data collected, giving the following results: the average time of the interruptions between compression cycles (STD 9.184 s, OTH 7.316 s, *p* < 0.001); the depth of compression 50–60 mm (STD 50.65%, OTH 60.22%, *p* < 0.001); the rate of compression 100–120/min. (STD 46.39%, OTH 53.78%, *p* < 0.001); complete chest wall recoil (STD 84.54%, OTH 91.46%, *p* < 0.001); correct hand position (STD 99.32%, OTH method 99.66%, *p* < 0.001). A statistically significant difference was demonstrated in the results to the benefit of the OTH method in the above parameters. The remaining parameters showed no significant differences in comparison to reference values.

**Conclusions:**

The higher quality of CPR in the simulated research using the OTH method by a single person justifies the use of this method in a wider range of emergency interventions.

## Background

High quality chest compression and artificial ventilation improves the effectiveness of CPR resuscitation and increases survival rates in patients with sudden cardiac arrest (SCA) [1–4]. The European Resuscitation Council (ERC) defines in its guidelines how CPR should be conducted [5]. Ensuring highly effective CPR can be particularly difficult when artificial ventilation using a bag-valve-mask device, combined with chest compression have to be carried out by one person [6]. This situation can occur in-hospital when, after beginning CPR, a member of medical staff has to wait for the resuscitation team, or out-of-hospital when a qualified person conducting CPR is waiting for the medical emergency team to arrive [7].

If a two-person medical emergency team is conducting advanced life support (ALS), difficulties can occur when one of the team is conducting chest compression and artificial ventilation using a bag-valve-mask device, while the other team member is carrying out other resuscitation actions such as defibrillation, treatment of reversible causes of SCA etc. [8, 9]. The ERC 2015 guidelines recommend conducting chest compressions from the patient’s side. Chest compressions from behind the patient’s head can be used, for example, during CPR in confined spaces [5].. The authors of a few studies have presented various CPR methods conducted by one person and assessed their effectiveness. The results obtained differ as regards key parameters in both chest compression and artificial ventilation [8–14].

Scientific research has shown that minimizing the interruptions between chest compressions during CPR has a direct influence on decreasing hemodynamic disorders, limiting the occurrence of complications, as well as increasing the effectiveness of CPR [15–19]. Interruptions during CPR can result from two ventilations, defibrillation or other resuscitation actions [5, 17, 18, 20].

In response to the doubts described above, assessment was carried out of the quality of CPR conducted by one paramedic in terms of chest compression and artificial ventilation from behind the patient’s head, in comparison to compression from the patient’s side with ventilation from behind the patient’s head.

## Methods

**Fig. 1:**
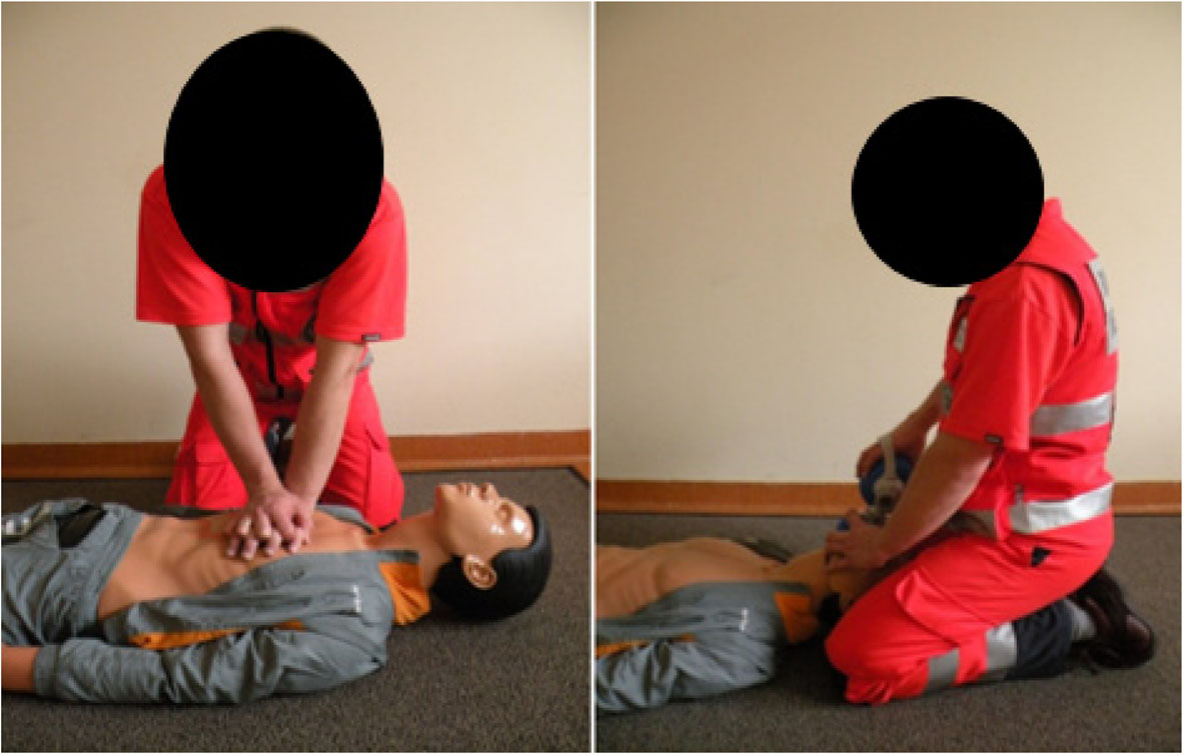
Chest compression and ventilation conducted using the ‘standard’ method. Own material

**Fig. 2:**
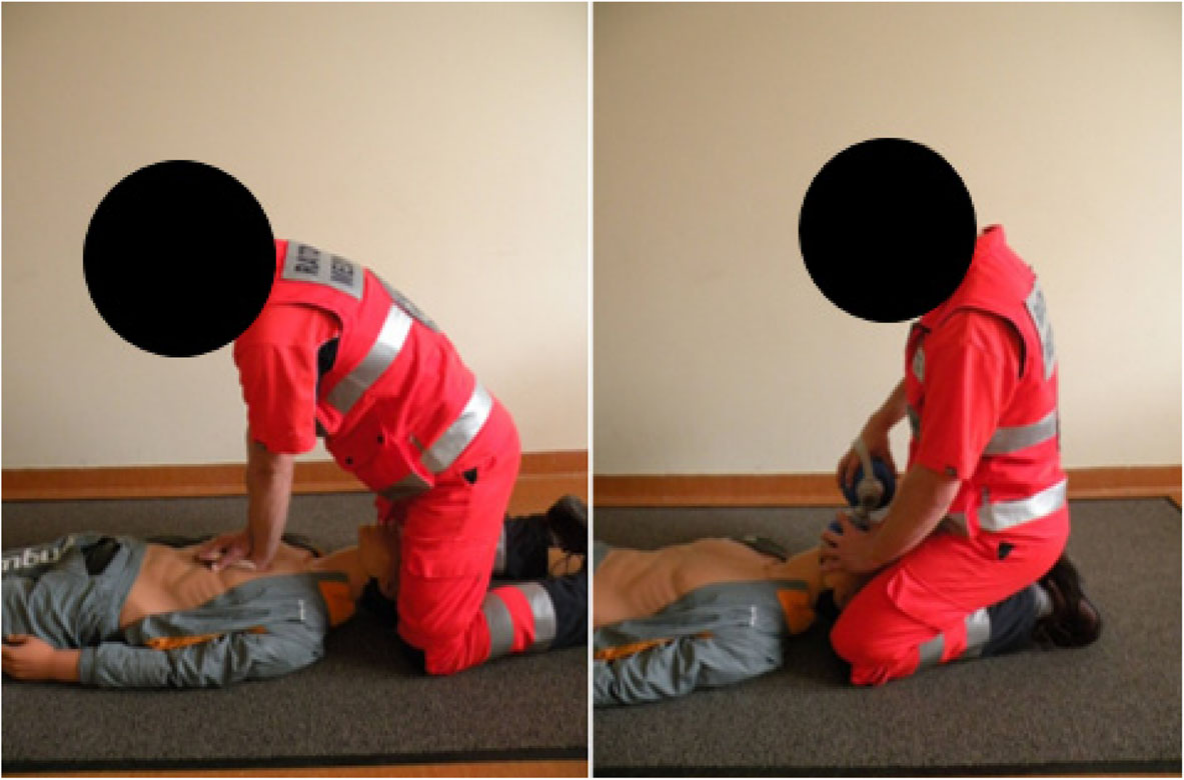
Chest compression and ventilation conducted using the ‘over-the-head’ method. Own material

The research was conducted in the Scientific Medical Emergency Laboratory of the Faculty of Health Science at the University of Bielsko-Biala. The subject of the study were two methods of CPR – ‘standard’ (STD) and ‘over-the-head’ (OTH). The STD method (Fig. 1) consisted of cycles of 30 chest compressions from the patient’s side, and then two attempts at artificial ventilation using a bag-valve-mask device after moving round to behind the patient’s head. To carry out the next 30 chest compressions, the paramedic returned to their position at the patient’s side. The OTH method (Fig. 2) consisted of cycles of 30 chest compressions from behind the patient’s head, and then two attempts at artificial ventilation using a bag-valve-mask device from the same position. Both CPR methods were carried out using an ‘Ambu Man W’ manikin, programmed with the parameters for an adult male with a body mass of 80 kg. Ventilation was carried out using a ‘Ambu Mark IV’ self-inflating bag. The research method consisted of analyzing CPR cycles (compressions/ventilation) carried out by research participants within a 5 min period.

The data collected from the manikin was sent directly to a computer with the Ambu CPR Software version 3.1.1. The software made it possible to assess the quality of chest compression and artificial ventilation for the parameters tested, as well as to define for them, based on ERC 2015 guidelines, the following reference values: the shortest possible pause time between chest compression cycles, the depth of chest compressions 50–60 mm, the rate of chest compressions 100–120/min, full chest recoil after each compression, compression in the centre of the chest (on the lower half of the sternum), duty cycle 50:50 (ratio of the time the chest is compressed to the total time from one compression to the next), the ratio of the number of chest compressions to the number of artificial ventilations 30 to 2, the volume of artificial ventilation conducted from 480 to 560 ml (for a patient with a body mass of 80 kg), without stomach inflation during ventilation.

Both CPR methods were carried out by members of medical emergency response teams. An advertisement was posted to which a group of men responded, from whom those qualified to take part in the research were selected based on inclusion and exclusion criteria. The following research qualification criteria were adopted: voluntary participation in the research, men and women, below 50 years old, people who had completed 3-year Bachelor’s studies in emergency medical response, obtaining the title of professional paramedic, at least 5 years’ experience working in a medical emergency response team, with theoretical knowledge and practical skills in conducting CPR in both the STD and OTH methods, current employment in the Bielsko-Biala Emergency Services, scoring at least 200 educational points during compulsory training sessions in a five-year study period, successful completion of a 48-h In-Service Training Course for Paramedics in a five-year study period. The regular Course currently follows standards of conducting CPR according to ERC guidelines. The exclusion criteria included: lack of professional experience in conducting chest compression from ‘over-the-head’, lack of permission for participation in the first and/or second stage of the research.

**Fig. 3:**
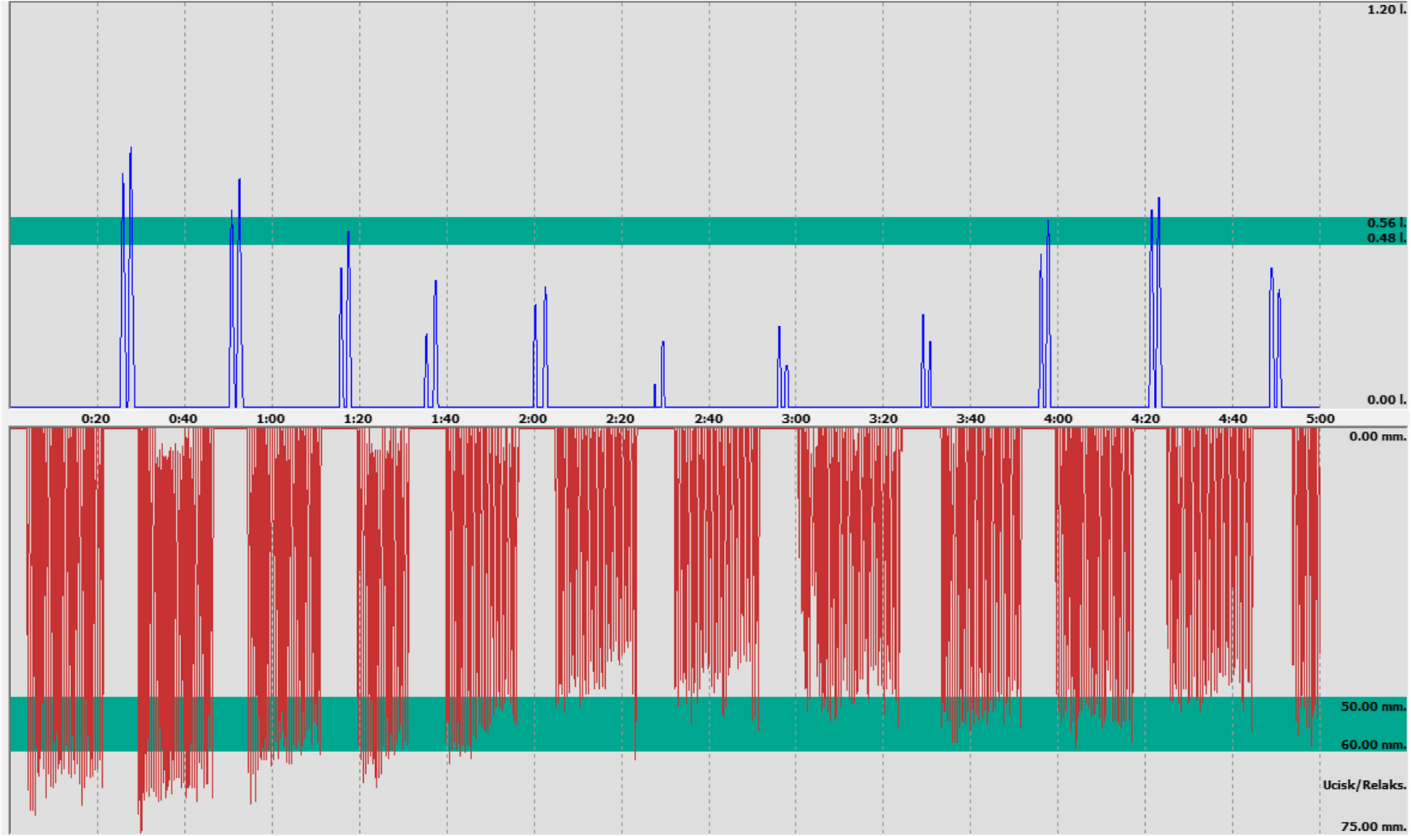
Example record of chest compressions and artificial ventilation (Paramedic no. 5 –OTH method)

At the first meeting between the research author and the participants, it was agreed that every participant would conduct CPR using both methods – STD and OTH. The research would be carried out in two stages with a 1 day break between the stages. The first stage consisted of: random selection by the research volunteers of the order of the methods for conducting CPR, a reminder for the participants by the research author of the techniques of cardiopulmonary resuscitation for the first of the drawn methods and the reference values for chest compression and artificial ventilation parameters based on ERC 2015 guidelines, trial chest compression and artificial ventilation conducted by each volunteer on a manikin “Ambu Man W” using the method drawn for 1 min. The actual study consisted of a participant conducting simulated CPR once on the manikin for 5 min using the first of the drawn methods. The second stage of the research consisted of: a reminder for the participants by the research author of the techniques of cardiopulmonary resuscitation for the second methods, and the reference values for chest compression and artificial ventilation parameters based on ERC 2015 guidelines, trial chest compression and artificial ventilation conducted by each volunteer on a manikin “Ambu Man W” using the second method for 1 min. The actual study consisted of a participant conducting simulated CPR once on the manikin for 5 min using the second of the drawn methods. Both stages were supervised by the author in terms of their compliance with the research assumptions. After the completion of both stages of the study, an evaluation was conducted of the results obtained for chest compression cycles and artificial ventilation with regard to the tested parameters. Figure 3 presents an example of a recorded chest compression and artificial ventilation cycle achieved by a single study participant.

The research results were recorded in such a way as to prevent identification of the participants according to applicable legal regulations. The research project received the approval of the Bioethics Committee in Bielsko-Biała. The results were compiled in a Microsoft Excel spreadsheet. The data was then imported into Statistica software manufactured by StatSoft (Ser.No:AXFP301D415123AR-B). To start with, basic statistical parameters were calculated for quantitative variables. Compliance tests were carried out for the normal distribution using the Kołmogorowa-Smirnowa test. For quantitative discrete (categorial) variables and qualitative variables, the distribution of abundance and percentage distributions for particular categories of these variables were determined. The basic criteria for the selection of the statistical method for data analysis were: variable type (qualitative, quantitative); ordinal, quotient and interval variables; compliance with the normal distribution; and skewness and curtosis values. Comparative analysis of the percentage fraction with regard to the cardiopulmonary resuscitation method was conducted using a structure index test. Evaluation of the interdependence of qualitative variables was conducted using Pearson chi-square tests of independence (χ^2^), or chi-square tests of independence (χ^2^ NW) using the highest credibility function. Comparative analysis of quantitative variables for when normal distribution and variance homogeneity conditions were met were conducted using parameter tests such as the Student’s t-test. In other cases, the Mann-Whitney U test and Wilcoxon test were used. Evaluation of the interdependence of quantitative variables for normal distribution was conducted using the Pearson correlation. For variables with a significantly different distribution to the normal distribution, the Spearman correlation was used.

## Results

The research was conducted in 2016 with the participation of 38 male paramedics employed in the Bielsko-Biala Emergency Services. The few women working in medical response teams did not volunteer to participate in the research. The paramedics participating in the research lived in the Bielsko-Biala region and had significant professional experience in advanced resuscitation techniques. Participants conducted both CPR methods under constant supervision by the research author, providing a guarantee of compliance with the conducting of the research and its assumptions.

The research results obtained were arranged into nine research parameters, taking into account the CPR method used and the range of reference values for individual parameters (Table 1).

**Table 1:** Collective summary of simulated CPR situation results for STD and OTH methods achieved by research participants

Parameters researched	Reference value	STD	OTH	Significance
Average time of interruptions between compression cycles [s]	Up to 10 s	9.184	7.316	*p* < 0.001
Depth of chest compressions	Too shallow (< 50 mm)	3898 27.80%	3103 20.77%	*p* < 0.001
	Correct (50–60 mm)	7104 50.65%	8997 60.22%	*p* < 0.001
	Too deep (> 60 mm)	3022 21.55%	2840 19.01%	*p* < 0.001
Rate of chest compressions	Too slow (< 100/min.)	57 12.84%	41 8.62%	*p* < 0.001
	Correct (100–120/min.)	206 46.39%	256 53.78%	*p* < 0.001
	Too fast (> 120/min.)	181 40.77%	179 37.60%	*p* < 0.001
Chest wall recoil	Complete recoil	11,856 84.54%	13,664 91.46%	*p* < 0.001
Place of chest compression	Centre of the chest	13,928 99.32%	14,889 99.66%	*p* < 0.001
Duty cycle (ratio of the time the chest is compressed to the total time from one compression to the next)	50%/50%	42.974% / 57.026%	42.237% / 57.763%	*p* > 0.05
Compression-ventilation ratio	30:2	78.51%	81.44%	*p* > 0.05
Tidal volume	Too low (< 480 ml)	459 51.37%	401 42.14%	*p* < 0.001
	Correct (480–560 ml)	152 17.05%	167 17.57%	*p* > 0.05
	Too high (> 560 ml)	282 31.58%	354 40.28%	*p* < 0.001
Stomach inflation	none	2 0.25%	3 0.34%	*p* > 0.05

The data presented shows that research participants conducting CPR using the OTH method more frequently achieved statistically significant (*p* < 0.05) shorter interruptions times between chest compression cycles than with the STD method.

In order to assess of the depth of chest compressions using the STD method compared to the OTH method, statistical analysis was conducted on the frequency of too shallow compressions (< 50 mm), correct compressions (50–60 mm) and too deep compressions (> 60 mm) taking into account the number of compressions made using a given method. It was shown that research participants conducting CPR using the OTH method more frequently made statistically significant correct depth compressions than with the STD method (*p* < 0.001).

Analysis of chest compressions cycles that were too slow (< 100/min.), correct (100–120/min.) and too fast (> 120/min.), taking into account the number of cycles conducted using a given method, showed that research participants conducting CPR using the OTH method more often made statistically significant correct rate chest compressions than with the STD method (*p* < 0.001).

Analysis of chest compressions with complete or incomplete recoil showed that research participants conducting CPR using the OTH method more often made statistically significant chest compressions with complete recoil than with the STD method (*p* < 0.001).

Assessment of the place chest compressions were made showed that research participants conducting CPR using the OTH method more often made statistically significant chest compressions in the correct place than with the STD method (*p* < 0.001).

Statistical analysis showed that the duty cycle in CPR using both the STD and OTH methods did not display a statistically significant difference (*p* > 0.05).

It was shown that research participants conducting CPR made cycles of chest compressions and the number of ventilations in the correct proportion 30:2, with the frequency showing no statistically significant difference between the OTH and STD methods (*p* > 0.05).

In order to assess the tidal volume of the artificial ventilation made using the OTH method compared to the STD method, analysis was made of ventilation with too low tidal volume (< 480 ml), correct tidal volume (480–560 ml) and too high tidal volume (> 560 ml). It was shown that there is no statistically significant difference in the frequency of correct tidal volume between the OTH and STD methods (*p* > 0.05). Research participants made a statistically significant higher number of ventilations with too low tidal volume when using the STD method than in the OTH method (*p* < 0.001). Research participants made a statistically significant higher number of ventilations with too high tidal volume when using the OTH method than in the STD method (*p* < 0.001).

Analysis of the frequency of stomach inflation showed that in research participants conducting CPR there were no statistically significant differences in ventilations with stomach inflation for both methods (*p* < 0.05).

## Discussion

The subject of numerous studies related to CPR has been the length of interruptions between chest compressions. Perkins et al. [5] claimed that effective treatment of patients with out-of-hospital cardiac arrest is dependent on the length of time between chest compressions necessary for the other resuscitation actions. Other authors [17, 19], including those conducting studies on an animal model [15], are of the same opinion, and indicate that any interruptions in chest compression, including those for artificial ventilation, have a significant negative effect on a patient’s chances of survival.

Our research has shown that the average length of time between chest compression cycles resulting from the necessity to conduct two attempts at ventilation is significantly shorter in the OTH method than when the STD method is used (*p* < 0.001). Similar results in studies using manikins were obtained by Maisch et al. [8] in their research comparing the effectiveness of CPR carried out using several methods. Our research shows that as a person conducting CPR using the OTH method is not required to change position, this significantly shortens the length of time between chest compressions, resulting in an increase in chest compressions applied compared to the STD method.

Irrespective of whether the person conducting chest compressions is at the patient’s side or behind their head, they must adjust the pressure on the sternum in order to achieve the correct depth of compression. This depth has been assessed in numerous scientific studies, both those conducted in clinical conditions as well as those conducted on laboratory animals [1, 21–23]. In their research, Maisch et al. [8] compared CPR conducted by paramedics from the manikin’s side and from behind the manikin’s head in terms of the depth of chest compressions. The author did not find significant differences between the frequency of insufficient and excessive chest compression depths and the reference values. The research results demonstrated that during ‘over-the-head’ CPR, when the paramedic does not change position, it is easier to adjust the pressure on the sternum to ensure that compressions of the correct depth are made.

The quality of CPR is affected by the rate with which chest compressions are made. Perkins et al. [5] recommend conducting chest compressions on adults at a rate of 100–120 compressions per minute. Scientific studies have proved that rates of compression of below 100/min. and above 120/min. result in a drop in cardiac capacity, and a subsequent increase in patient mortality [2, 24]. Our research has shown that in CPR conducted using the OTH method, chest compression cycles of the correct rate are made significantly more often than in the STD method. Chest compressions of insufficient and excessive rate occurred significantly more often in CPR cycles using the STD method.

During CPR, it is extremely important to conduct chest compressions when the chest is complete recoil. In ERC 2015 guidelines, Monsieurs et al. [25] recommend that the person conducting chest compressions should take care not to press on the chest in the relaxation phase, thus allowing the chest walls to return to their anatomical shape. Fried et al. [26] assessed cardiopulmonary resuscitation carried out on 108 patients with SCA. In the group studied, a total of 112,569 compressions were made, of which 12% were conducted without complete recoil. In our research, we analyzed chest compressions made with complete and incomplete recoil using the STD and OTH methods. It was shown that compressions with incomplete recoil were made significantly more often during chest compressions using the STD method.

An important factor that affects the quality of CPR is the place where chest compressions are made. In ERC 2015 guidelines, Perkins et al. [5] recommend conducting chest compressions in the centre of the patient’s chest to ensure quick identification of the correct place for compressions on the bottom half of the sternum. Our study has shown that a very small percentage of chest compressions were made in the incorrect place by research participants – in the ‘standard’ method 0.68%, and in the ‘over-the-head’ method 0.34% (*p* < 0.001). These results suggest that due to the lower percentage of compressions made in the incorrect place, using the OTH method can have a more beneficial influence on the hemodynamic effect of CPR than the STD method.

Based on clinical research and on animal models, it has been determined that the person conducting cardiopulmonary resuscitation should maintain the correct ratio of the time the chest is compressed to the total time from one compression to the next [5, 27, 28]. Our research showed no statistically significant differences for this parameter between the STD and OTH methods (*p* > 0.05).

Both ERC and AHA (American Heart Association) 2015 guidelines have demonstrated that during cardiopulmonary resuscitation, optimal blood flow through the organs is ensured by chest compressions and artificial ventilation at a ratio of 30 to 2 [5, 29]. The research showed that for both methods there was no significant difference in maintaining the recommended ratio of chest compressions to artificial ventilation.

Excessive or insufficient ventilation volumes can have negative consequences for a patient with SCA. ERC and AHA guidelines define the volume of air for artificial ventilation as 6–7 ml/kg of patient body mass [25, 30]. This value was adopted in our research as a ventilation volume reference parameter. It was demonstrated that the percentage of correct volume ventilations did not differ significantly in both methods (*p* > 0.05). Ventilation with insufficient volume was conducted significantly more often in the STD method (*p* < 0.001). Ventilation with excessive volume was conducted significantly more often in the OTH method (*p* < 0.001).

Conducting artificial ventilation can result in air finding its way into the stomach, causing regurgitation and inhaling of stomach contents into the airways. If the stomach is full of air, this can put pressure on the diaphragm and cause problems with ventilation. Research conducted on patients and on human corpses have confirmed the importance of reducing the frequency of ventilation and inspiratory pressure so as to minimize the risk of blowing air into the stomach during positive pressure ventilation in patients with unprotected airways [5, 31–33]. Our research showed that the percentage of artificial breaths where air found its way into the stomach did not differ significantly in both methods (*p* > 0.05).

For the following parameters: chest recoil, ratio of chest compressions to the number of ventilations, and stomach inflation during ventilation, comparison of the results obtained in both methods showed no statistically significant differences. Therefore, it can be assumed that in terms of the quality of resuscitation for these parameters, it is of no importance whether a paramedic conducts CPR using the STD or the OTH method.

## Limitations

The authors are aware that a certain limitation on the research is the fact that the study was conducted in simulated conditions on a small groups of participants. In order for the research to be used in clinical practice, it is planned to extend the research to a more numerous group of paramedics conducting CPR not only in the laboratory, but also in as realistic conditions as possible.

## Conclusions

Our research has shown that when CPR is conducted using the STD method, there can be significantly more difficulties in maintaining the correct resuscitation parameters than when applying the OTH method. The research results have shown that the STD method does not ensure significantly higher quality CPR for any of the designated ERC parameters. The values of five of the nine parameters tested are significantly closer to the reference values when using the OTH method. The higher quality of CPR in the simulated research using the OTH method by a single person justifies the use of this method in a wider range of emergency interventions.

## Data Availability

Please contact Michal Cwiertnia for data requests.

## Abbreviations

CPR: Cardiopulmonary resuscitation
STD: Standard
OTH: over-the-head
SCA: sudden cardiac arrest
ERC: European Resuscitation Council
ALS: advanced life support
AHA: American Heart Association
